# Betaine Supplementation Does not Improve Muscle Hypertrophy or Strength Following 6 Weeks of Cross-Fit Training

**DOI:** 10.3390/nu12061688

**Published:** 2020-06-05

**Authors:** Tatiana Moro, Francesca Badiali, Iader Fabbri, Antonio Paoli

**Affiliations:** 1 Department of Biomedical Science, University of Padova, 35100 Padova, Italy; tatiana.moro@unipd.it; 2 iF srl Faenza, 48100 Ravenna, Italy; francesca.badiali@gmail.com (F.B.); iader@iaderfabbri.com (I.F.)

**Keywords:** betaine, CrossFit©, strength, body composition

## Abstract

We aim to investigate the effect of 6 weeks of betaine supplementation on body composition and muscle performance during CrossFit© training. Twenty-nine subjects matched for training status (4.16 ± 0.95 day/week) and body fat mass (12.66 ± 4.08%) were randomly assigned to a betaine (BET; N = 14) or placebo group (PLA; N = 15). Body composition and cellular hydration were estimated with skinfolds measurement and bioelectrical impendence before and after 6 weeks of training. Muscle performance was assessed using three different tests: 3-RM back-squat for muscle strength, 2 km rowing test for aerobic capacity and Bergeron Beep Test for anaerobic capacity. Muscle strength assessed during back squat significantly increased in BET (*p* = 0.04) but not in the PLA group, however, there were no statistical differences between groups. Although not significant, fat mass was reduced in BET compared to PLA. Overall, body composition and cell hydration measurements did not change in response to training or betaine supplementation. Short-term (6 weeks) betaine supplementation supports muscle strength but was not ergogenic for trained subjects to aerobic and anaerobic performance in the CrossFit©-specific test.

## 1. Introduction

Betaine is a quaternary ammonium compound (1-carboxy-N, N, N-trimethyl-methanamine), first identified in sugar beet juice (Beta vulgaris). The molecule, present in both plant and animal, is a metabolite of choline and a substrate of one of the two metabolic pathways that converts homocysteine into L-methionine. Betaine plays a twofold role in human physiology: as a donor of methyl groups in the transmethylation of homocysteine and as an osmolyte for the osmotic balance. Betaine has hepatoprotective [1], metabolic [1,2] and cardiovascular [3,4] protective effects. Betaine is contained mainly in seafood, spinach and wheat germ or fibers, and on average its daily dietary intake is ~100–400 mg/day [5]. Unfortunately modern diets may lack betaine or, more in general, in other methyl donors [6], making the supplementation of these osmolytes an essential topic for human nutrition. Several pieces of research observed that a betaine supplementation of 2.5 to 5 g/day may be useful to improve body composition and muscle functions [7,8].

The mechanisms involved in the action of betaine on athletic performance are not fully understood, however they seem to be associated with its role in methylation. In particular, betaine can transfer its methyl group during the formation of methionine from homocysteine. Methionine contributes to the synthesis of creatine and carnitine [9,10], and thus serves as a contributor for energetic fuel. Aside from its metabolic role, methionine is also an important sulfur amino acid involved in protein turnover; the transamination promoted by betaine can provide more methionine and, as such, implement muscle growth and strength gains [11]. In addition, it seems that betaine can alter lactate levels, some substrates in the metabolism of fatty acids, and stimulate the insulin receptor and the autocrine/endocrine release of IGF-1. Recently, a study showed that in physically active subjects, the integration of 1.25 g of betaine for two weeks resulted in a significant increase in growth hormone (GH) and insulin-like growth factor (IGF-1), a decrease in cortisol levels, an increase in muscle protein kinase B (Akt) and of p70 S6 k [12]. This finding suggested that betaine may have a positive effect on both anabolic endocrine profile and the anabolic signal system, and thus may promote protein synthesis. Moreover, the molecule could be involved in the intracellular defense mechanisms and protection of the enzymes of the citric acid cycle, which tends to reduce their action in conditions of progressive dehydration and hyperthermia associated with the whole exercise. The osmoprotective effect of betaine seems to be exerted by a replacements of inorganic ions in the cells, which helps to preserve cell membranes and water homeostasis [13].

Betaine potential ergogenic property was first proposed by Borsook in 1952 [14], who observed a general increase in muscle strength and endurance after administering betaine in patients with poliomyelitis. Confirmations that betaine may be able to improve strength performance are limited and conflicting. For example, Hoffman et al. [15] observed an increased strength performance after 15 days of betaine supplementation, but the same authors failed to replicate these results on isokinetic force in a later study [16]. Another series of studies examined the effect of betaine ingestion during aerobic trials and showed a potential effect on anaerobic power outcomes [17], whilst others showed no effect on peak power output and total work during a similar exercise protocol [15,18].

CrossFit© is an increasingly popular training approach used for weight loss and muscle strength. CrossFit© is a high-intensity training which integrates rapid and strenuous ballistic movements, combining both aerobic and resistance workouts within and between each session. As such, CrossFit© requires high levels of aerobic capacity, muscle strength and power [19]. Often, this type of training takes place in a warm and not properly ventilated environment with an increased risk of dehydration. As a consequence, this kind of disciplines requires high peak power and resistance to fatigue to achieve better performance [20] and proper dietary supplementation may be beneficial for CrossFit© athletes.

Unfortunately, studies on ergogenic nutrients to enhance CrossFit© performance are still limited. In consideration of the above-described physiological effects of betaine, and the sport-specific metabolic characteristic of CrossFit©, it has been hypothesized that its supplementation may increase muscle performance and body composition in healthy trained CrossFit© athletes. The aim of this study was to analyze the effect of betaine supplement during 6 weeks of CrossFit© training. Our hypotheses were that compare to a placebo supplement, betaine would have a greater effect on muscle performance and intracellular hydration.

## 2. Materials and Methods

**Figure 1:**
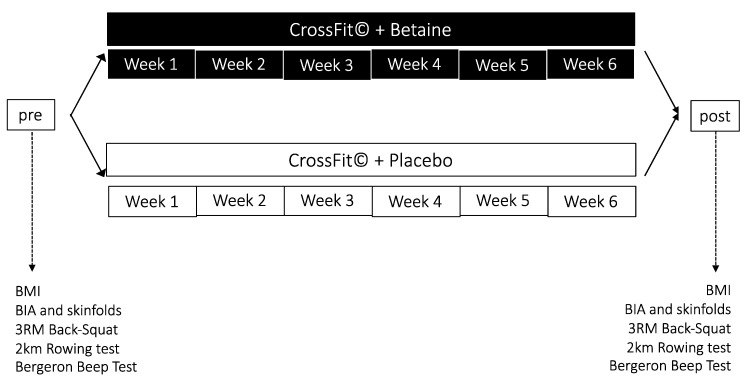
Study design.

This study is a randomized, double-blind design (Figure 1). After enrollment, body composition and physical performance were assessed in one sitting. Subjects were instructed to be 3 h fasted and to refrain from vigorous exercise, caffeine and alcohol for 24 h prior to being tested. Upon subject’s arrival, height and body weight were measured, followed by body composition assessment via Bioelectrical Impendence Analysis (BIA) and skinfolds. Physical performance tests were conducted immediately afterwards.

A computer-generated software (QuickCalcs, © 2018 GraphPad Software, San Diego, CA, USA) was used to randomly assign participants to two groups: Betaine supplementation (BET) or Placebo (PLA). Randomization was then checked to ensure that participants were pair-matched based on initial body fat percentage and strength levels. About one week later, participants received their intervention supplement supply and started the supervised cross-fit training period. Three days after the last exercise session, subjects repeated body composition and physical performance tests.

### 2.1. Subjects

Thirty-six subjects (age 35.11 ± 8.27; weight 72.03 ± 10.51 kg; BMI 24.18 ± 2.02 kg/m^2^) were screened and randomly assigned to BET or PLA group. Participants were recruited from three different gyms in the north of Italy. Subjects were included in the study if aged between 18 and 40 years old, with a BMI between 18.5 and 24.9 kg/m^2^, if they had at least 6 months of CrossFit^®^ experience and regularly engaged in physical training (>2 session/week). Thirty percent of the volunteers had more then 4–5 years of CrossFit^®^ background, 50% had been practicing for 2–3 years and the remaining 20% had 1–2 years of experience. Exclusion criteria were use of steroids, chronic use of medication or nutritional supplements, metabolic disorders or any clinical problems that could be aggravated by the study procedures. All participants read and signed an informed consent document with the description of the testing procedures approved by the ethical committee of the Department of Biomedical Sciences, University of Padova, and conformed to standards for the use of human subjects in research as outlined in the current Declaration of Helsinki. However, seven subjects were not compliant with the experimental procedures and were excluded from final analysis. In particular, five subjects did not show up for the post screening visit, and two subjects did not complete the performance test due to personal reasons. Thus, a total of 29 subjects were allocated in the BET (N = 14) and PLA (N = 15) group. The anthropometric baseline characteristics of subjects are shown in Table 1.

### 2.2. Measurements

Body weight was measured with a precision of 0.1 kg using an electronic scale (Tanita BWB-800 Medical Scales, Amsterdam, The Netherlands), and height to the nearest 1 cm using a wall-mounted portable stadiometer (Holtain Ltd., Crymych, UK). Body mass index (BMI) was then calculated in kg/m^2^. Body composition was assessed using triceps, abdominal and super-iliac skinfolds to the nearest 1 mm using a mechanical caliper (GIMA, Gessate MI, Italy) and fat mass and fat-free mass were estimated with the Jackson and Pollock equation [21]. As per standard procedures [22], all measurements were taken by the same operator (IF) before and after the study. Total body water (TBW), body cell mass (BCM) and phase angle (PA) were measured via Bioelectrical Impendence Analysis (INBODY, Caresmed S.r.l., Milano, Italy). Subjects were asked to void their bladder before the analysis and to lie down and rest for ~10 min, whilst two electrodes were placed on their hands and foots to start the test. Test–retest reliability for body composition obtained in our laboratory was consistent with previous findings: 0.99 for bioelectrical impendence analysis, and 0.96 for skinfold test [23].

Physical performance was assessed with three different typical CrossFit© exercises. A three-repetition maximum (3-RM) test during a back-squat exercise was used to assess leg muscular strength. After a proper warm-up, subjects performed five repetitions with a weight they normally lift 10 times. Using this as a starting point, intensity was gradually increased until subjects were able to perform only three repetitions with the assigned weight. The 3-RM is a reliable and safe test to assess muscle strength in both man end female; previously published ICCs for test–retest reliability 3-RM testing were 0.97 and 0.94, respectively [24]. Fifteen minutes later, aerobic performance was tested during a 2 km rowing test, in which subjects were asked to cover 2000 m in the shortest possible time using rowing equipment (Concept 2, Rogue Fitness). The test started with 3 min warm-up during which subjects performed 10 consecutive “hard” strikes at the start of each minute, as described elsewhere [20]. During the trial, subjects were encouraged to maintain the proper technique, with the movement starting from the leg, and followed by the back and arms. The time required to complete the task was recorded and compared to post-intervention results. Finally, 15 min later, subjects were asked to undergo to the Bergeron Beep Test, a multi-stage fitness test, employed to test anaerobic capacity. During the test, subjects were asked to perform seven thrusters, seven pull-ups and seven burpees in less than one minute and rest for the remainder of that minute. The test was ended when the participant was unable to complete a full round and the total number of repetitions performed was used as a score (i.e., if subjects quit at the end of the pull-ups during the third round, the final score was 56, (7 + 7 + 7) × 2 + 7 + 7). The test–retest reliability for 2 km rowing test and Bergeron Beep Test obtained in our setting was 0.95 and 0.90, respectively.

### 2.3. Supplementation

Treatments were administered in a double-blind fashion and the blind was removed after data analysis completion. Participants randomized in the BET group received 1.25 g of Betaine twice a day for a total of 2.5 g/day (1.25 g of betaine in 8 gr of microcrystalline cellulose). The PLA group received the same amount of inert compound (made of microcrystalline cellulose and flavors) to mimic the verum in appearance (size, colour, weight, feel, odour) of the treatment mixture. Both compounds were powdered, and had similar color and appearance; subjects were asked to dissolve them in ~100 mL of water before ingestion. The time of supplement ingestion was not strictly controlled, however subjects were asked to ingest their first dose in the morning and the second close to training session (within ~60 min from workout), and approximately at the same time during non-training days. Participants were given a weekly supply and a research team collected the empty supplement packets from each participant every week. Participants were also asked not to discuss their treatments with other volunteers.

The rationale behind the selection of 2.5 g/day as the treatment dosage is based on previous findings demonstrating that this is a sufficient dose to increase betaine plasma levels and to promote strength performance [25,26]. Other authors referred that a dosage of 34.8–36.3 mg/kg/LBM can improve power performance [15,27]; in the present study, the average was 43.2 mg/kg/LBM.

### 2.4. Training

Training was performed 2/3 time per weeks and each session lasted about 60 min. CrossFit^®^ is a codified brand (CrossFit, Inc., Washington, DC, USA) and is developed in workouts of the day (WODs) consisting of different exercises that combine gymnastics, weightlifting and metabolic conditioning. All sessions were performed at high intensity and rest periods between were minimal or absent. All training sessions were guided by a certified instructor and consisted of multi-joint exercises (such as skipping rope jump, wall ball shots, deadlift high-pulls, weightlifting, push presses, and rowing) combined in the form of a circuit [28]. Although the arrangement of exercise in each session might have varied between sites, all sessions were organized with a specific warm-up (~10 min) followed by skill technique workout (~10–15 min), the central phase of training consisted of a strength or conditioning workout (~30 min) and was concluded with mobility and cool down exercises (~5–10 min). According to the contents of the WODs, some workouts were aimed to perform as many rounds of the circuit as possible in a definite period of time or participants were asked to complete their routine in the best time possible. Maté-Muñoz has recently analyzed the three most common WODs and showed that all of them have a similar intensity and can be classified as “vigorous” exercise [28]. In the present study, subjects were asked to maintain their normal training routine, and thus we did not prescribe a unique and controlled training protocol; nevertheless, all participants had at least one year of experience and each subject served as control of themselves. This strategy permitted focusing on the effect of betaine supplementation without adding training as a new variable.

### 2.5. Statistical Analysis

Results are presented as mean ± standard deviation. Sample size was obtained assuming within-subject variability of 30% and a fixed power of 0.8 and an alpha risk of 0.05 for the main variables. Baseline differences between groups were tested with an independent samples t-test. After assessing the normal distribution with the Shapiro–Wilk W test, a parametric approach with a two-way repeated-measures ordinary ANOVA was performed (using time as the within-subject factor and supplement as the between-subject factor) in order to assess effect of Betaine over the course of the study. Sex differences at baseline and posttraining were tested with supplement x sex ANOVA. When the ANOVA model produced significant main or interaction effects, Bonferroni’s post hoc paired t-test was used to identify specific intragroup differences. Level of significance was set at *p* < 0.05. All analyses were done with GraphPad Prism 7.0 (GraphPad Software, La Jolla, CA, USA, https://www.graphpad.com/).

## 3. Results

Overall, women presented a significantly higher fat mass (BET 24.43 ± 2.89 vs. 15.04 ± 3.47 kg; PLA 23.96 ± 4.82 vs. 13.99 ± 2.98 kg) and lower free fat mass (BET 29.20 ± 2.47 vs. 42.14 ± 23.50 kg; PLA 28.94 ± 24.24 vs. 41.39 ± 23.53 kg). Women were also less performing compared to men on 3 RM squat test (BET 75.14 ± 15.92 vs. 116.82 ± 26.08 kg; PLA 68.00 ± 13.71 vs. 109.45 ± 18.78 kg); Bergeron Beep test (BET 67.14 ± 31.32 vs. 77.18 ± 37.38 reps; PLA 68.43 ± 39.13 vs. 87.00 ± 41.94 reps) and 2 km Row Test (BET 544.29 ± 37.77 vs. 470.91 ± 30.18 s; PLA 547.29 ± 36.02 vs. 486.36 ± 36.32 s). However, we didn’t detect any gender difference in response to training or supplement. For this reason, and considering that subjects’ gender was equally distributed between groups, we merged genders in the analysis.

There were no significant differences between groups in any variable. We observed a decrease of 1.35% of Fat Mass in the BET group and an increase of 2.12% in the PLA group, although this difference was not statistically significant (*p* > 0.05). Similar results were observed for Fat Free Mass (BET−0.25%; PLA−0.23%). Bioimpedance analysis showed a decrease in the phase angle in the BET group (−4.13%) and an increase in the PLA group (+5.28%). Total body water slightly increased in both groups (BET + 1.23%; PLA + 1.16%), and no difference was observed in the extracellular-intracellular water balance (*p* > 0.05). Data on body composition are presented in Table 2.

Table 3 shows the performance test results. After 6 weeks of training, we have not observed any significant time x training interaction; however, there was a main effect of time (*p* = 0.005) with BET group significantly improving back squat strength (+3.23%, *p* = 0.04) whilst the improvement in the PLA group was not significant (+2.05%, *p* = 0.13). Any significant difference was observed for the other variable: Bergeron Beep test (BET + 8.38%; PLA + 6.35%, *p* > 0.05) and 2 km row test (BET − 0.43%; PLA + 0.43%, *p* > 0.05).

## 4. Discussion

The main finding of the present study was that betaine supplementation supported submaximal strength but did not enhance strength endurance nor body composition after 6 weeks of CrossFit© training in experienced athletes.

Numerous studies conducted over the past decade suggest that health and optimal sport performance require an appropriate consumption not only of macronutrient (protein, carbohydrates and fat) but also of micronutrients (vitamins, minerals, etc.). Betaine is a methyl derivative of the amino acid glycine with three main functions in mammals: (1) shield cells under stress due to its osmolyte properties, (2) prevent protein structure degradation and (3) provide sources of methyl groups via transmethylation [7,29].

Animal and in vitro studies showed that betaine increases lean mass and reduces adipose tissue, probably due to a direct influence on growth hormones, IGF-1 expression, and protein synthesis regulation [30,31]. Moreover, the transmethylation of homocysteine to methionine via betaine, may promote protein synthesis due to the resulting methyl donation. Methionine is an essential amino acid for protein synthesis and its deficiency has a dramatic effect on whole-body protein turnover [11]. However, betaine can also indirectly affect muscle mass due to its organic osmolyte propriety, which creates an advantageous environment for the excitation contraction coupling and protein synthesis [32]. The first study conducted in humans was performed by Schwab et al. in 2002 [25] and failed to observe any alteration on body composition after 12 weeks of betaine supplementation in a group of sedentary obese adults. Later, Cholewa and colleagues observed that, when combined with exercise, betaine supplementation had a positive effect on lean mass and promote body fat decrease [8,27,33]. These contradictory results seem to suggest that exercise is an important factor to enhance betaine effect on body composition. However, in the present study, we did not observe any significant differences in lean or fat body mass between groups. Because the dose of betaine was similar to the one used in the above-mentioned studies (2.5 g/day), a possible explanation for the different results could be found in the training duration and intensity. Indeed, most of the studies on CrossFit© program required longer periods (>8 weeks) of treatment to observe significant changes in body composition [34,35]. Moreover, CrossFit© is a training approach characterized by the alternation of highly anaerobic and intense exercises, designed to increase muscle strength more than hypertrophy. It is plausible to believe that the combination of betaine with this specific exercise typology may not be successful to stimulate muscle growth. Although not significant, we observe a decrease in fat mass in the BET group compare to the placebo. Animals studies propose that betaine may stimulate lipolysis via GH release and by reducing triacylglycerol synthesis [7], it might be speculated that a longer period of treatment may have led to a more powerful effect on body fat mass.

Due to its osmolyte characteristic, betaine helps cell hydration and maintains cellular osmolarity. In vitro studies observed that, under osmotic stress, betaine administration prevents protein structural degradation and support protein synthesis redistributing the proper amount of water in the cell and ensuring an efficient biopolymer hydration [36]. We tested the effect of betaine on intracellular hydration via bioelectrical impedance. We did not observe any difference in intra- or extracellular hydration, nor in total body water due to betaine supplementation. Our results confirmed what was previously found by Cholewa and colleagues in young collegiate females [33]. Although subjects were instructed to maintain their regular nutrient intake, and to avoid food or liquid consumption before the analysis, it is possible that the uncontrolled diet may have influenced the results.

Creatine is the major consumer of methyl groups; for this reason it was proposed that betaine, through its transmethylation action, could enlarge muscle phosphocreatine stores and consequently improve muscle performance [15]. The first study that proposed an ergogenic role of betaine in muscle endurance and performance was conducted in patients with poliomyelitis in 1952 [14]. Later, Armstrong et al. [18] observed a positive effect on oxygen consumption and lactate concentration after betaine administration, results confirmed by others after high-intensity end strenuous endurance exercise [26,37]. It seems that betaine increase Krebs cycle function, reducing the thermodegradation of citric synthase and thus improving aerobic capacity [38]. Moreover, betaine supplementation seems to reduce the loss of muscle strength after fatiguing exercise [37] but its effect on strength boosting is still controversial. Only a few studies have investigated the longer effects of betaine supplementation on anaerobic muscle performance. Cholewa et al. [27] observed that after 6 weeks of resistance training combined with betaine supplementation, body composition, arm cross sectional area and strength were significantly increased. The authors suggested that betaine had an effect on training volume and quality, granted by an increased working capacity during the training sessions. Given the ergogenic potential of betaine on work capacity we hypothesized that 6 weeks of supplementation would have improved muscle performance during CrossFit© training, as this particular type of exercise requires strenuous and high-intensity exercises. We observed an improvement in the back-squat strength test in the BET group compare to placebo, whilst there were no significant differences in aerobic performance (2 km raw test) nor anaerobic capacity (Bergeron Beep test). This discrepancy with the previous studies can be found again on the nature of CrossFit© workouts. Indeed, we observed a positive effect of Betaine on the submaximal strength test, which use an anaerobic-phosphagen mechanism and is similar to the resistance training protocol studied by Cholewa at others in the abovementioned research. However, the energetic mechanism used during CrossFit© exercise is predominately anaerobic glycolytic (lactic acid). A study by Warren et al. [39] conducted in horses showed that betaine did not affect plasma lactate concentration during a an incremental test in trained animals. Thus, it could be possible that, due to the lactacid nature of CrossFit© exercise, the effect of betaine could not have implemented muscle performance because it does not interfere with lactate metabolism.

Moreover, subjects recruited for the present study had, on average, 2 years of training experience, and this may have impacted their ability to improve aerobic and anaerobic performance in such a short period of time (6 weeks). It seems that betaine may not directly influence lactate metabolism during exercise, but may help the release/uptake process during recovery period in untrained animals [39]. We can speculate that betaine supplementation may help to promote muscle performance during the adaptive phase of training but may not be advantageous in expert athletes.

Some limitations of the present study have to be considered. First, participants were asked to maintain their normal diet but without any restrictions. Thus, regular nutrient intake may have been different between subjects and might have influenced body composition. Secondly, subjects were asked to ingest the supplement daily, close to the training session but without a controlled timing. Betaine plasma concentration seems to be dose- and time-dependent: its absorption normally happens within 100–160 min, depending upon co-ingestion with other food. It could be plausible that a not-strict timing intake may have compromised its effect on muscle performance during training sessions, and by extension, also muscle size and strength. Another limitation may be represented by the fact that subjects were recruited in three different gyms, and thus functional test and training was supervised by different trainer. Although all trainers were certified and instructed on the study procedures, this may have affected the motivational status, and thus the results of performance tests. On the other hand, each gym recruited participants for both BET and PLA group, thus the effect of betaine supplementation should not have been altered by this limitation.

## 5. Conclusions

In conclusion, 6 weeks of betaine supplementation supports the increase in lower limb strength but does not enhance body composition, aerobic capacity or anerobic performance in trained CrossFit© athletes. We conclude that expert athletes may not benefit from betaine ergogenic proprieties due to their high training status, but it could be useful in novel athletes. Future studies comparing directly different training levels may be needed to address this question.

## Figures and Tables

**Table 1:** Baseline characteristics of Betaine (BET) and placebo (PLA) groups.

Variable	BET (N = 14)		PLA (N = 15)	
	Female (N = 7)	Male (N = 7)	Female (N = 7)	Male (N = 8)
Age (y)	36.29 ± 8.12	32.43 ± 8.24	34.86 ± 10.93	36.13 ± 8.69
Height (cm)	167.43 ± 4.08	174.50 ± 4.75	164.43 ± 8.83	178.25 ± 9.44
Weight (kg)	64.57 ± 7.57	78.37 ± 5.59	63.59 ± 13.82	76.29 ± 6.82
BMI (kg/m^2^)	23.01 ± 2.32	25.73 ± 1.28	23.25 ± 2.53	24.02 ± 1.45
Body Fat (%)	15.04 ± 3.47	24.43 ± 2.89	13.99 ± 2.98	23.96 ± 2.98
N° training/wk	4.14 ± 1.46	4.17 ± 0.75	3.71 ± 0.76	4.71 ± 0.76

All data are mean ± SD.

**Table 2:** Body composition analysis.

Variable	BET (N = 14)		PLA (N = 15)				
	Pre	Post	Pre	Post	Treatment *p*-Value (η²)	Time *p*-Value (η²)	Time x treatment *p*-Value (η²)
Fat Mass (%)	16.79 ± 7.06	16.57 ± 7.11	17.98 ± 9.02	18.20 ± 8.90	0.346 (0.067)	0.973 (0.001)	0.336 (0.001)
Fat Free Mass (%)	59.53 ± 10.65	59.47 ± 11.14	57.77 ± 12.49	57.63 ± 12.35	0.187 (0.126)	0.520 (0.001)	0.883 (0.001)
Phase angle (°)	8.02 ± 1.09	7.66 ± 0.99	6.74 ± 2.70	8.11 ± 1.39	0.255 (0.040)	0.131 (0.031)	0.085 (0.087)
Active cellular mass (kg)	36.00 ± 7.64	35.89±7.62	35.89 ± 7.79	36.15 ± 8.08	0.832 (0.003)	0.959 (0.001)	0.082 (0.001)
Total Body Water (L)	41.14 ± 7.11	41.68 ± 7.62	40.99 ± 8.32	40.61 ± 8.41	0.831 (0.004)	0.743 (0.001)	0.720 (0.001)
ECW/ICW (%)	0.67 ± 0.05	0.67 ± 0.06	0.66 ± 0.05	0.66 ± 0.06	0.514 (0.030)	0.793 (0.001)	0.745 (0.001)

Data are mean ± SD. ECW = Extracellular Water; ICW = intracellular water.

**Table 3:** Performance test results.

Variable	BET (N = 14)		PLA (N = 15)				
	Pre	Post	Pre	Post	Treatment *p*-Value (η²)	Time *p*-Value (η²)	Time x Treatment *p*-Value (η²)
Back Squat (kg)	95.08 ± 35.91	98.08 ± 36.79 *	88.86 ± 27.60	91.00 ± 29.63	0.062 (0.294)	0.005 (0.009)	0.230 (0.001)
Bergeron Beep test (reps)	74.25 ± 34.68	71.33 ± 25.96	73.77 ± 35.03	73.77 ± 33.16	0.394 (0.059)	0.772 (0.001)	0.255 (0.011)
2 km Row (s)	526.27 ± 42.91	523.36 ± 35.09	501.00 ± 38.45	503.08 ± 40.38	0.547 (0.039)	0.906 (0.001)	0.488 (0.007)

Data are mean ± SD. * significantly different from pre-value (*p* < 0.05).
